# Dr. Robert V. K. Montfort

**Published:** 1901-02

**Authors:** 


DR. ROBERT V. K. MONTFORT, of Newburgh, N. Y., died in that city December 29, 1900, aged 65 years. He was an alumnus of Albany Medical College, '59, served as a medical officer during the civil war with distinction, was formerly health officer of Newburgh, and was a prominent educator, having served many years as clerk of the board of education and superintendent of public schools of Newburgh.

